# Quantification of Arm Swing during Walking in Healthy Adults and Parkinson’s Disease Patients: Wearable Sensor-Based Algorithm Development and Validation

**DOI:** 10.3390/s20205963

**Published:** 2020-10-21

**Authors:** Elke Warmerdam, Robbin Romijnders, Julius Welzel, Clint Hansen, Gerhard Schmidt, Walter Maetzler

**Affiliations:** 1 Department of Neurology, Kiel University, Arnold-Heller-Straße 3, 24105 Kiel, Germany; r.romijnders@neurologie.uni-kiel.de (R.R.); j.welzel@neurologie.uni-kiel.de (J.W.); c.hansen@neurologie.uni-kiel.de (C.H.); w.maetzler@neurologie.uni-kiel.de (W.M.); 2 Faculty of Engineering, Kiel University, Kaiserstraße 2, 24143 Kiel, Germany; gus@tf.uni-kiel.de

**Keywords:** gait, gyroscope, inertial measurement unit, Parkinson’s disease

## Abstract

Neurological pathologies can alter the swinging movement of the arms during walking. The quantification of arm swings has therefore a high clinical relevance. This study developed and validated a wearable sensor-based arm swing algorithm for healthy adults and patients with Parkinson’s disease (PwP). Arm swings of 15 healthy adults and 13 PwP were evaluated (i) with wearable sensors on each wrist while walking on a treadmill, and (ii) with reflective markers for optical motion capture fixed on top of the respective sensor for validation purposes. The gyroscope data from the wearable sensors were used to calculate several arm swing parameters, including amplitude and peak angular velocity. Arm swing amplitude and peak angular velocity were extracted with systematic errors ranging from 0.1 to 0.5° and from −0.3 to 0.3°/s, respectively. These extracted parameters were significantly different between healthy adults and PwP as expected based on the literature. An accurate algorithm was developed that can be used in both clinical and daily-living situations. This algorithm provides the basis for the use of wearable sensor-extracted arm swing parameters in healthy adults and patients with movement disorders such as Parkinson’s disease.

## 1. Introduction

A distinct feature of human locomotion is the rhythmic swinging motion of the arms [1,2]. The amplitude of the swing is associated with gait speed and cognitive loading [3,4]. Active increase of arm swings has the potential to stabilize gait [5]. The reduction of arm swing amplitude and other alterations of the arm swing pattern, including asymmetry and irregularity, can be related to neurological pathologies. In stroke patients, the arm swing amplitude of the affected arm is smaller compared to that of the controls [6]. Patients with Parkinson’s disease (PwP) also show a smaller arm swing amplitude and, in addition, more asymmetry, compared to controls [7,8,9]. Therefore, the arm swing is regularly evaluated in a clinical setting and has the potential to improve diagnostic accuracy [7,10,11] and map disease progression [7,11]. Asymmetry in PwP might be associated with disease progression, as a study with 16 PwP in an early disease stage reported a positive correlation between asymmetry and the Hoehn and Yahr (HY) stage in an off-medication state [12]. Similar results were observed in eight mild PwP, showing a positive correlation between asymmetry and the Unified Parkinson’s Disease Rating Scale (UPDRS) of the limbs [7]. However, another study analyzed 21 PwP with HY stage I and 19 PwP with HY stage II using an ultrasound-based motion analysis system, and the study found more asymmetry in the HY stage I PwP group compared to the HY stage II PwP group [8]. Levodopa intake or dopaminergic treatment has shown to improve arm swing amplitude, peak swing velocity, and asymmetry of the amplitude in 104 moderate to severe PwP [13]. This was confirmed for asymmetry in another study investigating 16 mild to moderate PwP [12].

Due to the dynamic technical development, the measurement of human movement and mobility has been revolutionized over the last decades and years. Wearable inertial systems (inertial measurement units, IMUs) are an especially attractive assessment tool for arm swings, as these techniques make it possible to measure movements during everyday life [10,14,15,16]. The relevance of measuring mobility in everyday lives of patients is increasingly recognized because it is likely to differ substantially from the mobility that is performed in front of a healthcare professional [17].

This study presents, to our best knowledge for the first time, the technical development and clinical validation of a wearable sensor-based arm swing algorithm for healthy adults and PwP.

## 2. Materials and Methods

### 2.1. Subjects and Data Collection

There were 15 healthy adults and 14 PwP who participated in this study. The study was approved by the ethical committee of the medical faculty of Kiel University (D438/18) and performed in accordance with the Declaration of Helsinki of 1975. All subjects provided written informed consent before participating. The inclusion criterion for the healthy adults was no disorders that affect movement, and the inclusion criterion for PwP was a Parkinson diagnosis according to UK Brain Bank Criteria [18].

The healthy subjects walked at three different speeds (2, 3, and 4 km/h) on a treadmill (size: 2.2 by 0.7 m; Woodway, Weil am Rhein, Germany) for 80 s. The PwP walked on their self-selected speed on the same treadmill for at least 60 s.

### 2.2. Definition of Arm Swing during Locomotion

**Figure 1:**
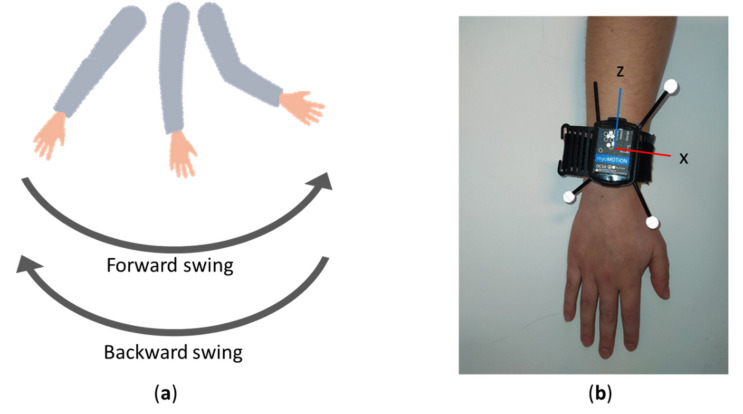
(**a**) Definition of swings. (**b**) Placement and orientation of the right-handed coordinate system of inertial measurement unit and reflective markers.

In order to develop this algorithm, it was necessary to define the movement “arm swing” in such a way that on one hand it is coherent with existing information [1,2], and on the other hand also addresses the characteristics of the technology used. We therefore propose the following definition:

**Definition** **1.** *Arm swing is a rotational movement of the arm, occurring during walking and running in bipeds with a periodicity of around 1–2 Hz. The hand and arm move freely through space in opposite directions with most of the movement in the sagittal plane of the body frame (backward and forward; Figure 1a).*

This arm swing algorithm was developed for the data collected during walking. The periodicity of an arm swing had to be between 0.3 and 3 Hz. The minimum amplitude to define an arm swing was set at 5°. Only rotations around the frontal and sagittal axis were taken into account because the wearable sensor might not always be aligned with the sagittal plane of the body frame during the swinging motion of the arms. In this way, all the rotations of the arms are measured except the longitudinal rotations, since they will also be influenced by turns of the body.

### 2.3. Equipment

All subjects were equipped with a cluster of three reflective markers (11 mm) and an inertial measurement unit (IMU) (Noraxon USA Inc., Scottsdale Arizona, AZ, USA) containing 3D accelerometers, 3D gyroscopes, and 3D magnetometers, on each forearm. The position of the markers was aligned with the position of the IMUs to have a similar orientation of the right-handed coordinate systems (Figure 1b). The markers were captured with a 3D optical motion capture system (Qualisys AB, Göteborg, Sweden) at 200 Hz. Both systems recorded simultaneously at 200 Hz.

### 2.4. Data Processing

#### 2.4.1. Inertial Measurement Unit Data

Only the gyroscope data of the IMU were used in this offline algorithm. The algorithm was written with MATLAB 2017a.

The gyroscope data were filtered with a zero-phase second order Butterworth low pass filter with a cut off frequency of 3 Hz to omit noise and possible tremors ($\omega_{filt}$). A principal component analysis (PCA) was performed on the x and y component of the angular velocity. The longitudinal component (*z*-axis) was not taken into account for the PCA in order to remove any longitudinal rotations (such as turning) from the data. From here on, only the first component of the PCA ($\omega_{PCA1}$) is used for the analysis. This first component represents the angular velocity in the direction of the arm swing. Extracting the angular velocity in the swing direction makes this algorithm insensitive to different wearing locations of the IMU on the forearm as long as the *z*-axis is aligned with the longitudinal axis of the arm. The angle (α) was calculated from the angular velocity in the swing direction ($\omega_{PCA1}$) by numerical integration using a trapezoidal integration approximation:

(1) $$\alpha \left(t\right)=\int_{\tau =0}^{t}\omega_{PCA1}\left(\tau \right)d\tau .$$

A symmetric moving average (${\hat{m}}_{\alpha }$) was calculated with a window length of 2q+1, where q is half a second (representing a window length of 1.005 s with a sample frequency of 200). The moving average was subtracted from the angular data to remove the low frequency drift.

(2) $${\hat{m}}_{\alpha }\left(n\right)=\;\sum_{j=-q}^{q}b\left(j\right)\;\alpha \left(n+j\right),\;q<n<N-q;\;\mathrm{with}\;b\left(j\right)=\;\left\{\begin{array}{ll} \frac{1}{4q}, & if\;j=\pm \;q\\ \frac{1}{2q}, & else \end{array}\right.$$

(3) $$\alpha_{detrend}\left(t\right)=\;\alpha \left(t\right)-\;{\hat{m}}_{\alpha }\left(t\right).$$

The frequency was extracted with a fast Fourier transform (FFT) from 3 s rectangular windows with 75% overlap. The dominant frequency was extracted from each window. The percentage of the power that was in the 0.3–3 Hz domain was calculated and used to determine whether there was a periodical movement in this specific frequency domain of arm swing motion. When this percentage was below an empirically determined threshold of 90%, this window was not taken into account for further analysis.

**Figure 2:**
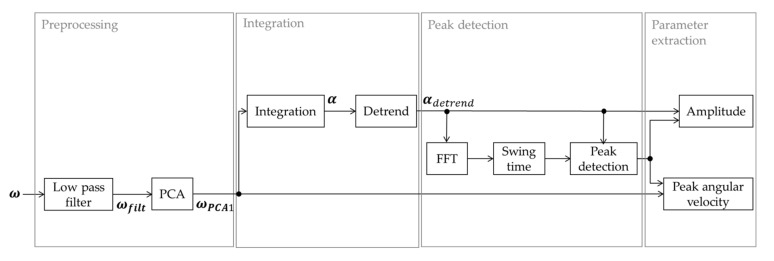
Block diagram of the arm swing algorithm.

The local maxima and minima from the angle signal ($\alpha_{detrend}$) were extracted. Both the positive and negative peaks needed to have a minimum peak prominence of 2° and a minimum distance of 60% of the cycle time that was extracted from the dominant frequency per window from the FFT. The overlap of the 3 s rectangular windows for the peak detection was 50%. Peaks that were detected multiple times due to the overlapping windows were only considered once. In between two maxima, only one minimum was allowed, and in between two minima only one maximum was allowed. In case of an extra detected peak, the smallest peak was discarded. The magnitudes of a consecutive minimum and maximum or a maximum and minimum were added to each other to obtain the amplitude of the swing. The time instants of these extrema were then used to find the extrema in the angular velocity in the swing direction to obtain the peak angular velocity. When a swing took longer than twice the average cycle time, it was discarded because of the low probability of it being an actual arm swing. Any outliers (peaks that were larger than three times the 80th percentile of the peaks detected in the angle signal) were removed because those were probably other movements than the regular swinging motion during walking (e.g., scratching the head). Every swing with an amplitude below 5° or a peak angular velocity below 10°/s was removed from the data because a high detection accuracy cannot be guaranteed during such small arm movements. An overview of the main steps taken are provided in Figure 2.

Additionally, the peak angular velocity was divided into forward and backward angular velocities, based on whether it was a minimum or a maximum in the angular velocity in the swing direction. This makes it possible to analyze potential differences caused by the direction of the movement. When there were no periodical movements of the arm or the arm movements were too small, no arm swing parameters were calculated. To understand whether the amplitude and peak angular velocity were calculated during the complete walking bout or only for a shorter period, the percentage of time in which there were swings detected in one arm during the walking bout was extracted. How frequently the arms moved was represented in the frequency as was extracted with the FFT. The similarity between neighboring swings was represented with the regularity. The regularity was calculated based on the autocorrelation of the angle [19]. The autocorrelation was extracted with a 4.5 s Tukey window with a cosine fraction of 0.3 and a 99% overlap of the windows. The maximum autocorrelation of each window was extracted, and the average of these values was taken as regularity. A regularity of 1 means that a swing is exactly similar to its neighboring swings.

When both arms were measured and the IMUs were synchronized, the percentage of simultaneously occurring arm swings in both arms was calculated. Arm swings were deemed simultaneous when a change in direction (i.e., forward to backward or backward to forward) of an arm swing in one arm was within 500 ms from a change in direction of the arm swing in the other arm. If at least 60% of the walking episode was with simultaneously swinging arms, the asymmetry index (ASI) was calculated for the average amplitude and peak angular velocity. For the calculation of the ASI, only the phases with swings detected in both arms simultaneously were taken into account [20]:

(4) $$ASI=\;\frac{\left(L-R\right)}{\max\left(L,\;R\right)}\;\times 100$$

where *L* is the amplitude or the peak angular velocity of the left arm and *R* the similar parameter of the right arm. An ASI of 0% reflects identical values of the left and right arm. The coordination between the left and right arm was calculated when during at least 60% of the walking episode, arm swings were detected in both arms simultaneously. The coordination was based on the normalized cross-correlation of which the minimum value was calculated. The absolute of this minimum was calculated for each swing during the phases where there were arm swings in both arms simultaneously, of which then the average was taken to obtain the coordination. This is a slightly adjusted version of [12], where they calculated the maximum of the absolute signal instead of the absolute minimum.

(5) $$r_{LR}\left(m\right)=\;\frac{\sum_{n=0}^{N-m-1}\omega_{PCA1\_L}\left(n+m\right)\;\omega_{PCA1\_R}\left(n\right)}{\sqrt{\sum_{n=0}^{N-m-1}\omega_{PCA1\_L}{\left(n\right)}^{2}\;}\sqrt{\sum_{n=0}^{N-m-1}\omega_{PCA1\_R}{\left(n\right)}^{2}\;}},$$

(6) $$coordination=\frac{1}{n}\sum \left|\min\left(r_{LR}\left(m\right)\right)\right|.$$

with $\omega_{PCA1\_L}$ and $\omega_{PCA1\_R}$ the angular velocity in swing direction of the left and right arms respectively, and m ranging from 0 ± 0.5 s. A value of 1 indicates that the left and right arms swing with a similar rhythm that is exactly out of phase with each other. A value of 0 indicates that there is no coordination between the arms.

The algorithm is available online (https://github.com/EWarmerdam/ArmSwingAlgorithm).

#### 2.4.2. Optical Data

Gaps in the optical data smaller than 250 ms were filled based on marker intercorrelations [21]. The parts of the data with gaps larger than 250 ms were discarded. A local coordinate system was calculated from the three markers on the wrist. The angular velocity was obtained from the derivative of the orientation. The orientation was also used to calculate the Cardan angles (order: zxy). The angle and angular velocity were rotated in the swinging direction based on the results from the PCA of the IMU data. From there on, the amplitude and peak angular velocity were obtained in the same way as with the IMU data.

### 2.5. Statistical Analysis

For the validation, the data of both arms were taken together. To compare the angle and the angular velocity between both systems, the root mean square errors (RMSe) between the IMU and the optical data were calculated. A Bland–Altman analysis was performed to extract the systematic error (average of the difference between the IMU-derived and the optical system-derived data) and the random error (95% confidence intervals ± systematic error) of the arm swing amplitude and the peak angular velocity [22]. The average absolute error was calculated to obtain the magnitude of the error between the two systems.

For the clinical validation, the arm swing parameters of the healthy participants walking at different speeds were compared to those of the PwP group. The amplitude, peak angular velocity, percentage of walking bout with arm swing, frequency, and regularity were calculated with averaged data of the left and right arms. The percentage of the walking bout with the arm swing in both arms simultaneously, asymmetry, and coordination were calculated by comparing left versus right arm data. For the asymmetry, the magnitude was taken for the analysis. A Mann–Whitney U test was used to test for significance (*p* < 0.05).

## 3. Results

One PwP was taken out of the analysis because all amplitudes of the arm movements did not reach the 5° threshold. An overview of the remaining participants taken into the analysis is provided in Table 1.

### 3.1. Healthy Adults

**Figure 3:**
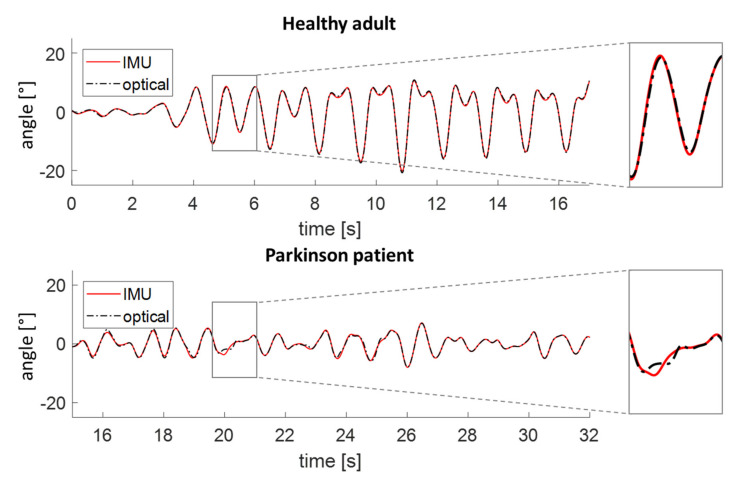
The angle of the inertial measurement unit (IMU) and optical data of a healthy participant and of a patient with Parkinson’s disease.

**Figure 4:**
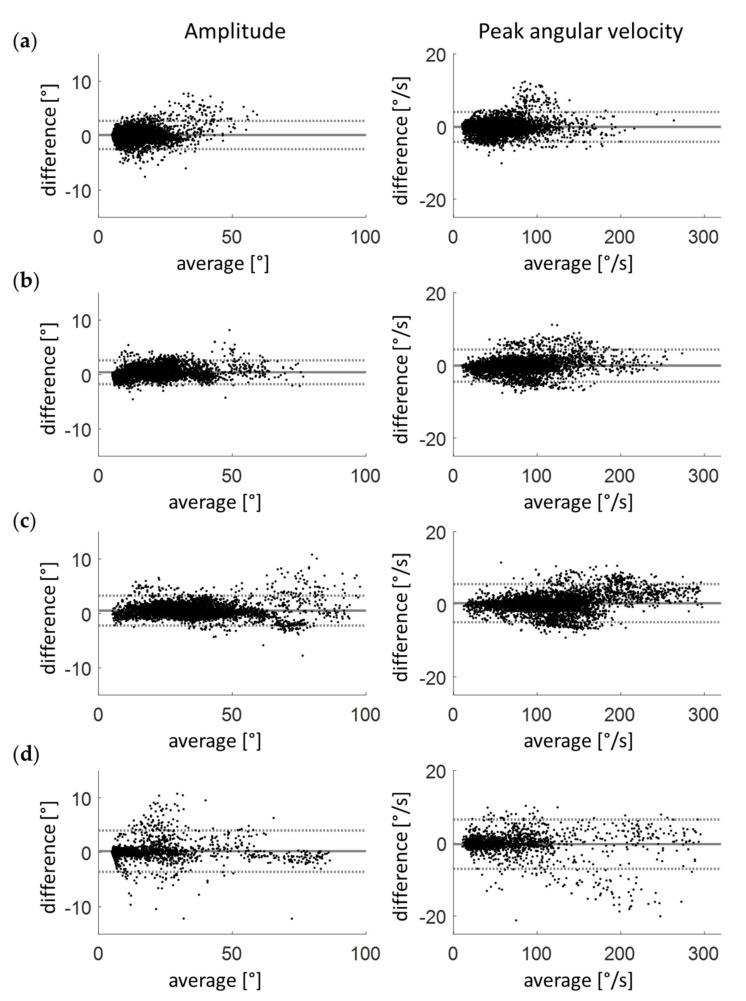
Bland–Altman plots are shown with the arm swing amplitude and peak angular velocity at 2 km/h (**a**), 3 km/h (**b**), and 4 km/h (**c**) for the healthy adults and at the preferred speed (**d**) for patients with Parkinson’s disease. On the x-axes, the average of the IMU and optical results are presented, and on the y-axes the differences between IMU and optical results (IMU-optical) are presented.

Fifteen healthy adults walked at three different speeds on a treadmill. The RMSe of the angle and angular velocity between the IMU- and optical system-derived signals were below 1° and below 0.05°/s, respectively (Figure 3, Table 2). The systematic errors were in the range of 0.1 to 0.5° for the amplitude and −0.1 to 0.3°/s for the peak vertical velocity of the different speeds (Figure 4, Table 2). The random error of the amplitude was between 2.2 and 2.7°, and the random error of the peak angular velocity was between 4.2 and 5.3°/s. The absolute errors ranged from 0.9 to 1.1° for the amplitude and from 1.4 to 1.9° for the peak angular velocity.

### 3.2. Patients with Parkinson’s Disease

Thirteen PwP walked at their preferred speed (average 1.4 km/h) on a treadmill. The RMSe between the IMU-derived and optical system-derived data was 1.16° for the angle and 0.16°/s for the angular velocity (Figure 3, Table 2). The systematic errors were 0.2° and −0.3°/s for the amplitude and peak angular velocity, respectively (Figure 4, Table 2). The random errors were 3.8° and 6.8°/s, and the absolute errors were 1.1° and 2.0°/s for the amplitude and peak angular velocity, respectively.

### 3.3. Clinical Validation

All the arm swing parameters were extracted with the algorithm and compared between the groups. The percentage of the walk with swinging motion in one arm was the only parameter that was significantly different between the groups on all speeds. On higher speeds, more significant differences were found between the groups (Table 3).

## 4. Discussion

This study presents the development and the validation of an arm swing algorithm based on wearable sensors (i.e., IMUs) positioned on the wrists for healthy adults and PwP. Based on our data, the algorithm is extremely accurate. Arm swing amplitude and peak angular velocity can all be extracted with a very small systematic error compared to the reference system.

The random errors are slightly higher for the PwP group compared to the healthy adults group. This may—at least partly—be due to the less fluent movement of the arms in PwP. It can be seen in Figure 3 and in the RMSe (Table 2) that the IMU and optical data do not overlap as well in the PwP compared to the curves derived from a healthy adult. This deviation between the IMU and optical data is especially seen around the peaks.

The healthy adults were measured at multiple speeds. Based on visual interpretation, the walking speed was not of influence on the accuracy of the algorithm. This should make the algorithm suitable for measuring arm swings in usual daily-living situations, which is particularly relevant for longitudinal and therapy studies. However, the algorithm itself cannot detect when someone is walking and might therefore include other repetitive movements of the arm that are performed throughout the day. Ideally, the arm swing algorithm should therefore be combined with a gait detection algorithm [23,24] when used for measurements outside the lab to make sure as much as possible that arm swings are only analyzed during walking. It should also be noted that a walking bout needs to be at least 3 s for the algorithm to work. For daily-living assessments, a higher minimum walking bout length might need to be set to exclude artefacts. This can omit wrongly increased variability of the data. Users of the algorithm should also take arm swing data from longer walking bouts with a certain degree of caution, as also during such walking episodes, arm movements that are not arm swings as defined in the introduction can occur. Examples are arm movements that are not based on freely moving hands (e.g., when swinging a bag or using Nordic walking sticks) and animated movements (e.g., performed based on a given rhythm that comes from earphones of external sources).

According to the protocol of a future study or the main objectives of clinical management that aim to integrate this algorithm in their approaches, the algorithm may be adapted to individual needs and situations. For example, in this particular study, arm swings with an amplitude below 5° were excluded. This is a very low threshold (corresponding to a horizontal displacement of 6 cm with an arm length of 70 cm), and can lead to false positive results in less strictly defined data sets (for example, it may detect movements of the arms and hands that are in the pockets during walking). Therefore, for daily-living assessments, we suggest increasing the threshold for the amplitude and combining it with a gait detection algorithm. Future studies must evaluate which thresholds have the highest accuracies, especially when recording unsupervised daily-living data. It should be mentioned again that this inaccuracy falls within the clinical and phenomenological domain and does not call into question the high technical validity of the algorithm (i.e., the compliance with the reference; see above).

For an initial clinical validation, all the parameters from the algorithm were extracted and compared between healthy adults and PwP. The percentage of the walk with swinging motion of the arms was significantly different in PwP, compared to all walking conditions performed with healthy adults. This makes a comparison of the arm swings between the groups difficult because we have to assume that in the PwP group, those arm swings are exactly the ones not included in the calculation that fall below the specified threshold of 5°. Therefore, the following qualitative comparisons must be interpreted with caution. Nevertheless, differences can be found in all group comparisons (Table 3).

When we compared the 4 km/h condition of the healthy adults, which comes probably closest to their preferred speed, we found significant differences in arm swings between the groups, and this finding corresponds to the literature [25,26,27]. Since we found less significant differences on 2 km/h, it could be that walking speed has an influence on the differences found between healthy adults and PwP, which certainly has to be investigated in future studies.

The lateralization of the disease may also have a relevant influence on arm swing parameters in PwP. A study with slow walking speeds on a treadmill only found significant differences for the amplitude between the most affected side of PwP compared to healthy adults [8]. Our results on asymmetry corroborate these preliminary results. The percentages of the walks with simultaneously performed swinging motions in both arms were substantially lower in PwP, compared to healthy adults at all measured walking speeds. We assume similarly according to our reasoning in the above paragraph that all qualitative evaluations that were performed in the PwP group may thus underestimate the real asymmetry and lack of coordination of arm swings because it is exactly those arm swings with high asymmetry and low coordination values that are excluded based on our threshold (arm swing > 5°). Nevertheless, it is noticeable (see also Table 3) that PwP have higher amplitude and peak angular velocity asymmetry indices than healthy adults. In conclusion, our preliminary clinical results indicate that the known differences in arm swing between PwP and healthy adults can be reliably and accurately detected with this algorithm, and future clinical studies may include this algorithm.

A study reporting about prodromal changes of gait in PD was recently published [28], but it did not report about arm swing behavior. The algorithm presented here can now be used to analyze such data sets with higher granularity and more exhaustive information about body movement. The algorithm can also extend the movement assessment for observational studies, clinical trials, and clinical management to the daily-living environment, an area that we have not been able to investigate and understand in much detail so far. The evaluation of disease progression and response to treatment in PwP has a similar or even higher relevance, not only for the amplitude of arm swings but also for all other parameters presented in Table 3. Arm swing parameters could help to differentiate healthy adults from PwP, and they may be useful for the detection and diagnosis of additional diseases associated with impaired mobility (such as multiple sclerosis). Of course, the application of this algorithm also opens up new options in the evaluation of arm swings in the context of aging in general, with respect to the significance of arm swings in fallers, and how arm swings differ between supervised and unsupervised environments, to name a few examples.

Some aspects should be taken into account when using the algorithm in future studies. First, turns during walking in daily living have no influence on the algorithm itself, since rotations around the longitudinal axis are not taken into account. When the walking turns should be separated from the walking data, a turning algorithm should be used to detect the turns [29,30]. Second, in general, the arm moves in phase with the contralateral leg. However, on slower speeds, the arms can swing in a 2:1 ratio with the legs instead of 1:1 [31,32]. This in itself is no issue for the algorithm. However, during the transition phases between these two ratios (Figure 3, about 7 s), it depends on how fast the frequency changes and whether the swing is above the set thresholds if this swing in between is detected. When it is detected, it might influence the variance of the data, since the amplitude, peak angular velocity, and average angular velocity are smaller compared to the other swings. Third, people can be measured on one or two wrists. It is self-explanatory that in case of only one wearable device, the percentage time where there was a swing in both arms, the asymmetry, and the coordination cannot be calculated. Fourth, for some of the PwP, there were only a few arm swings detected during the walking bout because the arm movements did not exceed the 5° threshold. This is likely to happen more often in severe PwP.

The study faces the limitation that during the measurements the participants walked on a treadmill, which results in slightly different upper body movements compared to over ground walking [33]. However, we consider this a minor issue, as the main aim of the study was the validation of the IMU-derived arm swing algorithm against a reference that was assessed simultaneously. Moreover, the healthy controls were in their young adulthood and thus substantially younger than PwP. This implies that we are mapping an age effect in the clinical validation data for which we cannot correct in this data set. However, we are optimistic that we will still map a Parkinson-associated difference, as our data confirm the data from previously published studies. We are also working on a detailed representation of arm swings in existing data sets of large cohorts, including the TREND study (https://www.trend-studie.de/).

## 5. Conclusions

An arm swing algorithm was developed and validated for both healthy adults and PwP. The algorithm is highly accurate in a clinical environment and has high potential to be used in a daily-living environment as well.

## Figures and Tables

**Table 1:** Demographics (mean ± standard deviation) of the subjects.

	Healthy Adults	PD Patients
n (male)	15 (9)	13 (5)
Age [years]	31 ± 9	71 ± 9
Body mass index [kg/m^2^]	23.4 ± 2.7	28.5 ± 5.9
Hoehn and Yahr stage (1–5)	NA	2.8 ± 0.7

**Table 2:** Error measures of IMU-derived arm swing data, compared to optical system-derived data.

		Healthy Adults 2 km/h	Healthy Adults 3 km/h	Healthy Adults 4 km/h	PwP Preferred
Angle RMSe [°]		0.83	0.91	0.72	1.18
Angular velocity RMSe [°/s]		0.03	0.03	0.03	0.16
No. of swings		3885	3788	4103	1762
Amplitude [°]	Systematic error	0.1	0.4	0.5	0.2
	Random error	2.6	2.2	2.7	3.8
	Absolute error	0.9	0.9	1.1	1.1
Peak angular velocity [°/s]	Systematic error	−0.1	−0.1	0.3	−0.3
	Random error	4.2	4.4	5.3	6.8
	Absolute error	1.4	1.6	1.9	2.0

PwP: patients with Parkinson’s disease; RMSe: root mean square error.

**Table 3:** IMU-based arm swing parameters for the healthy adults and the patients with Parkinson’s disease.

	Healthy Adults (2 km/h)	Healthy Adults (3 km/h)	Healthy Adults (4 km/h)	PwP (Preferred)
Amplitude [°]	16	23 *	36 *	17
Peak angular velocity [°/s]	57	84 *	122 *	60
Forward peak angular velocity [°/s]	59	87 *	124 *	60
Backward peak angular velocity [°/s]	55	80 *	120 *	59
Percentage of walk with swinging motion in an arm [%]	93 *	99 *	99 *	78
Frequency [Hz]	0.9	0.9	0.9	0.9
Regularity (0–1)	0.8	0.9 *	0.9 *	0.7
Percentage of walk with swinging motion in both arms simultaneously [%]	90 *	97 *	98 *	64
Absolute amplitude asymmetry index [%]	20	17	20	36
Absolute peak angular velocity asymmetry index [%]	19	18	21	33
Coordination (0–1)	0.7	0.8	0.8	0.8

*: significantly different from patients with Parkinson’s disease (*p* < 0.05); see the data processing part in the methods for the calculations and interpretation of the parameters. For the asymmetry and coordination, seven PwP could be included in the analysis; the other four did not fulfil the criteria for the calculation of these parameters (see Methods section).
